# Potential therapeutic effect of *Allium cepa* L. and quercetin in a murine model of *Blomia tropicalis* induced asthma

**DOI:** 10.1186/s40199-015-0098-5

**Published:** 2015-02-21

**Authors:** Tatiane Teixeira Oliveira, Keina Maciele Campos, Ana Tereza Cerqueira-Lima, Tamires Cana Brasil Carneiro, Eudes da Silva Velozo, Ingrid Christie Alexandrino Ribeiro Melo, Eugênia Abrantes Figueiredo, Eduardo de Jesus Oliveira, Darizy Flávia Silva Amorim de Vasconcelos, Lain Carlos Pontes-de-Carvalho, Neuza Maria Alcântara-Neves, Camila Alexandrina Figueiredo

**Affiliations:** Instituto de Ciências da Saúde, Universidade Federal da Bahia, Salvador, Bahia Brazil; Faculdade de Farmácia, Universidade Federal da Bahia, Salvador, Bahia Brazil; Centro de Biotecnologia, Universidade Federal da Paraíba, João Pessoa, Paraíba Brazil; Centro de Pesquisas Gonçalo Moniz, Fundação Oswaldo Cruz, Salvador, Bahia Brazil

**Keywords:** Natural product, Asthma, *Blomia tropicalis*, *Allium* cepa L., Quercetin

## Abstract

**Background:**

Asthma is an inflammatory condition characterized by airway hyperresponsiveness and chronic inflammation. The resolution of inflammation is an essential process to treat this condition. In this study we investigated the effect of *Allium cepa* L. extract (AcE) and quercetin (Qt) on cytokine and on smooth muscle contraction *in vitro* and its therapeutic potential in a murine model of asthma.

**Methods:**

AcE was obtained by maceration of *Allium cepa* L. and it was standardized in terms of quercetin concentration using high performance liquid chromatography (HPLC). *In vitro*, using AcE 10, 100 or 1000 μg/ml or Qt 3.5, 7.5, 15 μg/ml, we measured the concentration of cytokines in spleen cell culture supernatants, and the ability to relax tracheal smooth muscle from A/J mice. *In vivo*, *Blomia tropicalis* (BT)-sensitized A/J mice were treated with AcE 100, 1000 mg/kg or 30 mg/kg Qt. We measured cell influx in bronchoalveolar lavage (BAL), eosinophil peroxidase (EPO) in lungs, serum levels of Bt-specific IgE, cytokines levels in BAL, and lung histology.

**Results:**

We observed a reduction in the production of inflammatory cytokines, a relaxation of tracheal rings, and a reduction in total number of cells in BAL and EPO in lungs by treatment with AcE or Qt.

**Conclusion:**

AcE and Qt have potential as antiasthmatic drugs, as they possess both immunomodulatory and bronchodilatory properties.

## Background

Asthma is an inflammatory condition characterized by airway hyperresponsiveness, mucus cell hyperplasia, inflammatory cell infiltration and reversible bronchoconstriction, which may progress to airway remodeling with fibrosis and an increase in smooth muscle reactivity [1-4]. In allergic asthma, exposure to allergens causes an imbalance between the T helper type (Th) 1 and Th2 responses. Cytokines produced by the Th2-type CD4 + T cells (interleukin (IL-4, IL-5, IL-13) in asthma have a central role in orchestrating the inflammatory response. Strong support for this T-cell-centric paradigm has been enriched by the identification of Treg cells with the capacity to control both Th1 and Th2 responses [5]. The synthesis and release of IL-4 (which stimulates B cells to synthesize IgE), IL-13 (which stimulates mucus production) and IL-5 (which is necessary for eosinophilic infiltration to the lung tissue) increase vascular permeability and chemotaxis, which amplify the inflammatory response. Additionally, activated mast cells are able during an allergen challenge to release several inflammatory and bronchoconstrictor mediators [6,7].

The prevalence of asthma continues to rise worldwide [8], and treatment of asthma faces many challenges, including under diagnosis, access to care, ability of health-care workers to manage asthma, education of healthcare providers and patients, and availability and affordability of inhaled therapy [9]. Treatment with inhaled steroids and bronchodilators often results in good control of symptoms [10]. However, the treatment for patients with severe asthma with uncontrolled and frequent exacerbations still contributes to morbidity and mortality of asthma in all age groups and remains a challenge [11,12]. The safety concerns and the obstacles for the asthmatic patients justify continued efforts to find new alternative therapies [13].

Historically, herbal medicine has been studied in asthma treatment, and some of the drugs currently used to treat this disease such as the inhaled corticosteroids, sympathomimetics, anti-cholinergics, methylxanthines and cromones have origins in herbal treatments [14]. Thus, our group performed an ethnopharmacological survey [15], and one of the herbs described was *Allium cepa* L., commonly used to treat inflammatory conditions such as asthma [16,17].

Several plant-derived secondary metabolites have been shown to interfere directly with molecules and mechanisms, such as the mediation of inflammatory responses and activity of second messengers, as well as the expression of transcription factors and key pro-inflammatory molecules [18]. The main compounds found in *Allium cepa* L. extract (AcE) are the flavonoids such as quercetin, which are natural phenolic compounds present in fruits and vegetables, exhibiting many pharmacological properties such as its anti-inflammatory and antioxidant effects [17,19-21]. Along with flavanols, the major bioactive constituents in *Allium cepa* L. are sulfurous compounds. In previous studies, using mass spectrometry for direct analysis of volatile sulfurous compounds has described the presence of propanethiol, dipropyl disulfide and thiosulfinates [22-24]. The sulfoxides, which are responsible for the onion flavor and odor, might also be responsible in part for the onion biological activity of different *Allium* spp. species. The propanethiol is suggested to be the main source of the characteristic onion odor [23,24].

In previous studies, the anti-allergic potential of the extracts of *Allium cepa* L. [16,17] and its flavonoid quercetin [18,19,25] has been reported using a mouse model of ovalbumin (OVA)-induced asthma. This study is the first which was conducted using extracts of *Allium cepa* L. (AcE) and quercetin treatment in murine model of allergic airway disease induced by the sensitization to the clinically relevant aeroallergen *Blomia tropicalis* mite. This mite is a major house dust mite in dust worldwide [26]. In addition, it has been shown that flavonoids, typically found in *Allium cepa,* have a relaxing effect on the smooth muscle of isolated trachea and may have bronchodilator effect [25,27,28].

Thus, the objective of this study was to assess the therapeutic potential (anti-inflammatory and bronchodilator) of the methanolic extract of *Allium cepa* L. (AcE) and its flavonoid quercetin (Qt) in a murine model of respiratory allergy to *Blomia tropicalis* mite.

## Methods

### Animals

A/J mice aged 5–7 weeks (20–25 g), females, were from the Fundação Oswaldo Cruz, Bahia, Brazil. Five animals per group were used in each experiment. Mice were housed in controlled temperature and humidity environment with 12-hour light–dark cycles, and had free access to food and water. The experimental procedures were approved by the Ethical Committee for Use of Experimental Animals of the Faculdade de Odontologia, Universidade Federal da Bahia, Brazil (protocol number: 02/09).

### Sensitization and challenge of mice with *Blomia tropicalis* (Bt-sensitized mice)

**Figure 1:**
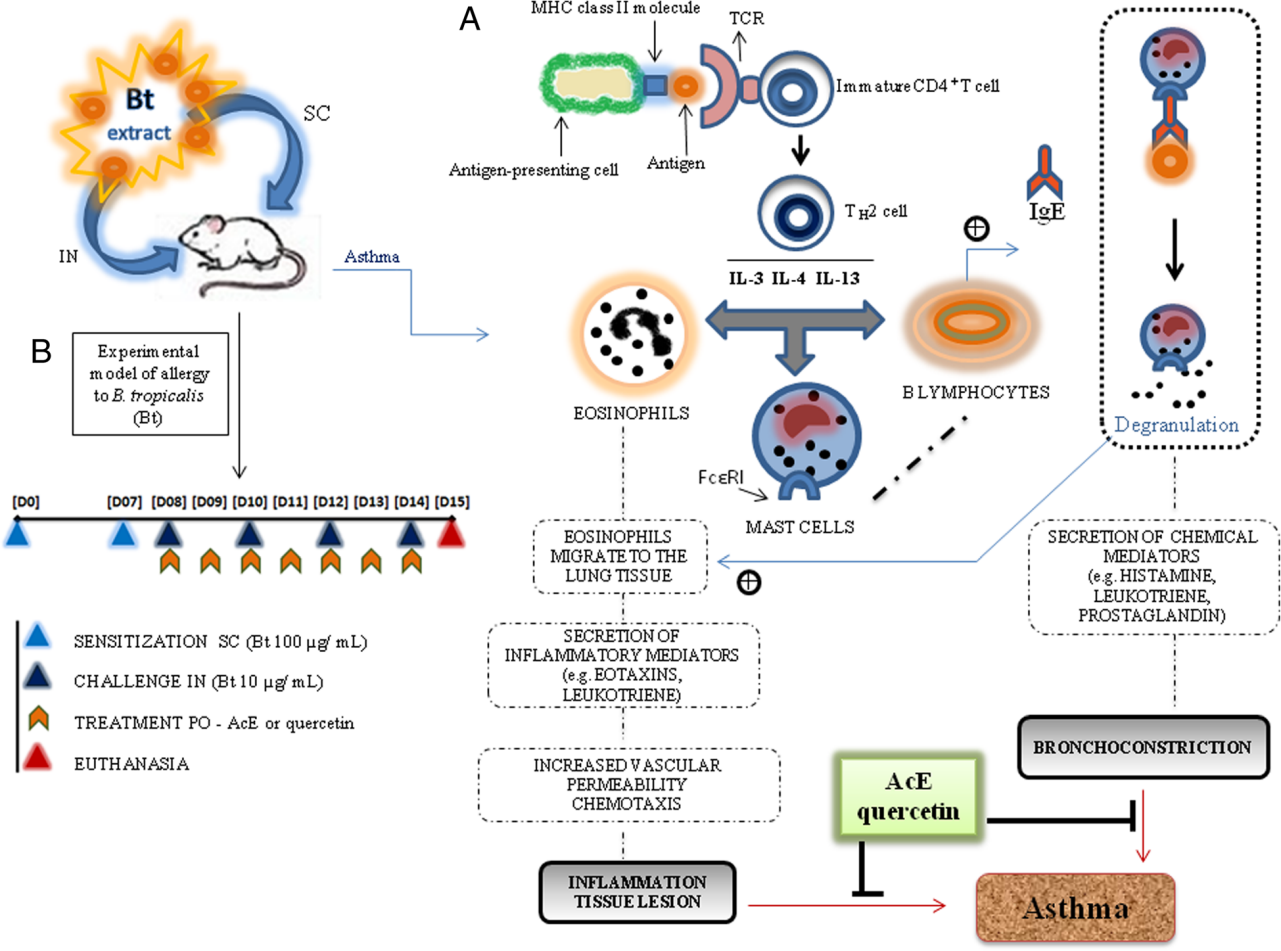
**Representation of the asthmatic response (A) and experimental design of allergy to** ***B*** **.** ***tropicalis*** **(Bt) in B.**

A *B. tropicalis* extract was obtained as previously described [15]. The experimental model of allergy to *B. tropicalis* (Bt) dust mite was used as previously described [29]. The animals were sensitized with subcutaneous injections of Bt (100 μg of protein) adsorbed to 4 mg/ml of Al(OH)_3_ in saline on days 0 and 7, and 1 day after the last sensitization the animals received four intranasal challenges with Bt (10 μg/instilation) every other day. Animals were euthanized with intraperitoneal injections of xilazine and ketamine (40 mg/kg/body weight), 24 hours after the last challenge. A schematic diagram of the sensitization and allergen challenge schedule is shown in Figure 1.

### Preparation of the *Allium cepa* L. extract and obtaining of quercetin

A methanolic extract was obtained by maceration of *Allium cepa* L. previously peeled and cut in a closed container containing 1000 ml of 99.8% methyl alcohol (CH_3_OH) during 7 days at room temperature. Subsequently, we proceeded to filtration of the extractive liquid, which then was rota-evaporated at a temperature of 60–70°C in a thermostatic bath. This concentration process was repeated three times. The AcE was dried in the oven and then maintained at −20°C until use.

The flavonoid quercetin [2-(3, 4-dihydroxyphenyl)-3, 5, 7-trihydroxy-4H-1-benzopyran-4-one, 3, 3′, 4′, 5, 6 - entahydroxyflavone] was obtained commercially (98% purity) from Sigma-Aldrich® (St. Louis, MA, USA).

*In vitro*, different concentrations of AcE—10 μg/ml (AcE_10_), 100 μg/ml (AcE_100_), or 1000 μg/ml (AcE_1000_), and Qt—3.5 μg/ml (Qt_3.5_), 7.5 μg/ml (Qt_7.5_) or 15 μg/ml (Qt_15_) were tested. *In vivo*, the tested groups were animals sensitized to Bt and daily treated orally with AcE containing 100 mg/kg (AcE_100_) and 1000 mg/kg (AcE_1000_) or 30 mg/kg Qt (Qt_30_) dissolved in saline (vehicle) from the 8th to the 14th day of the experimental protocol and one hour after the intranasal challenges with Bt. Mice receiving the following treatments were studied: non-sensitized and vehicle-treated mice (negative control group); Bt, Bt-sensitized mice and vehicle-treated mice (positive control group); Bt/AcE_100_, Bt-sensitized and AcE_100_-treated mice; Bt/AcE_1000_, Bt-sensitized and AcE_1000_-treated mice; Bt/Qt, Bt-sensitized and Qt_30_-treated mice (tested groups).

### Standardization of *Allium cepa* L. extract

The major flavonoid found in *Allium cepa* L. is quercetin [30,31]. The AcE was standardized in terms of quercetin concentration by high performance liquid chromatography, with ultraviolet light detection at 250 nm, using a C-18 column (150 × 4.6 mm ID, 5 μm particle size, Thermo Scientific) and a C-18 pre-column (Phenomenex, Torrance, USA). The mobile phase consisted of 0.1% aqueous formic acid (A) and methanol (B) at a flow rate of 1.0 ml/min. The following gradient elution method was used for separation: 5% to 90% of B in A in 30 min. The injection volume was 20 μL and quercetin calibration curves were in the range of 5 to 100 μg/ml.

### Cell viability assay

Cell viability after *in vitro* exposure to AcE and Qt was determined using the MTT assay. Spleen cells were seeded at a density of 5 × 10^5^ cells/well in 96-well plate and were then exposed to AcE and Qt diluted in RPMI. After 48 hours of incubation, the viability of the cells was evaluated by using FCS-free medium containing 1 mg/ml of 3-[4, 5-dimethylthiazol-2-yl]-2, 5 diphenyltetrazoliumbromide (MTT, Sigma). After 4 hours of incubation at 37°C, the medium was discarded and the formazan blue dissolved with DMSO. The optical density (OD) was measured at 540 nm. The percentage of viable cells was calculated by defining the cell viability without treatment as 100%.

### *In vitro* cytokine production by spleen cells

Levels of IL-4, IL-5 and IL-13 T-helper (Th) type 2 cytokines in splenocytes cultures were evaluated. According to the method described by Bezerra-Santos [32], splenocytes from Bt-sensitized mice were washed twice in RPMI medium by centrifugation at 200 × g for 10 min. The obtained pellet was resuspended in RPMI medium supplemented with 200 mM l-glutamine, 100 units/ml penicillin, 100 μg/ml streptomycin, 5β-mercaptoethanol and 10% fetal calf serum (Gibco, Pisley, UK). Viable cells number was determined in a hemocytometer by exclusion using trypan blue. The spleen cells were plated in 96-well flat-bottomed tissue culture plates (Costar, Cambridge, MA, USA) at a density of 5 × 10^5^ cells/well. The cells were treated with non-cytotoxic concentrations of AcE or Qt and stimulated with 5 μg/ml of pokeweed (PWM) (Sigma Aldrich, Saint Louis, MA, USA). The cells were incubated at 37°C in a humidified atmosphere of 5% CO_2_ for 48 hours. Supernatants of the cell cultures were collected and analyzed by enzyme-linked immunosorbent assay (ELISA) for IL-4, IL-5 and IL-13 cytokine concentrations (BD Pharmingen, San Diego, CA, USA).

### *In vitro* airway smooth muscle relaxation

AJ mice were euthanized and the trachea was rapidly removed. The trachea was cleared of loose connective tissue and divided into 2 rings of 2 mm, containing on average three to four cartilage bands. The rings were suspended on metal rods, attached to a force transducer (FORT10 WPI, Sarasota, USA) and placed in tanks for isolated organ with Krebs-bicarbonate solution (composition in mM: NaCl 119, NaHCO_3_ 25, CaCl_2_ × H_2_O 1.6, KCl 4.7, KH_2_PO_4_ 1.2, MgSO_4_ × 7H_2_O 1.2 and glucose 11.1), aerated with a carbogen mixture (95% O_2_ and 5% CO_2_) and maintained at 37°C. After the stabilization period (1 hour at 0.5 g), the rings were contracted with 10 μM of carbachol (Cch; Sigma, St Louis, MA, USA) to assess the contractile state of the tissue and to evaluate the presence of functional epithelium. The rings, after reaching a plateau of contractile state, were stimulated with bradykinin (Bk; Sigma, St Louis, MA, USA) (10^−6^ M). The rings were again contracted with Cch (10 μM) and cumulatively increasing concentrations of AcE or Qt were added. Concentration response curve was constructed and data were analyzed. Additionally, the tracheal rings were previously exposed to IL-13 in culture as described previously [33] to evaluate the effects of AcE or Qt on the hyper-responsive airway smooth muscle. The rings were placed individually in each well of a 48-well plate and incubated at 37°C in the presence or absence of IL-13 (10 ng/ml, 24 hours; BD Pharmingen, San Diego, CA, USA) in supplemented Dulbecco’s modified Eagle’s medium (containing 25 mM D-glucose, 1 mM sodium pyruvate, 100 U/ml penicillin, 100 μg/ml streptomycin, 0.2 M L-glutamine, 2.5 μg/ml Fungizone, and 0.1% w/v bovine serum albumin) (Sigma, St Louis, MA, USA) for 24 hours. Cumulative concentration-response curves to Cch after incubation in the absence or presence of IL-13 was built to analyze the hyper-reactivity of smooth muscle. After that, concentration-response curved for AcE or Qt was built.

### Collection of bronchoalveolar lavage (BAL) fluid and cell counting

The trachea of the euthanized mice were canulated and BAL fluid was obtained by three successive aspirations (total volume 1.5 ml) via tracheal cannulation of phosphate buffered saline, pH 7.4 (PBS) containing 1% of bovine serum albumin (Sigma-Aldrich St. Louis, MA, USA). Total number of leukocytes in the BAL was determined using Trypan blue and differential cell counts for eosinophils were performed by using Wright-stained cytospin preparations. Differential counts of eosinophils of at least 100 cells were made in a blind fashion in accordance with standard morphologic protocol. The concentrations of IL-4, IL-5 and IL-13 in BAL were quantified by ELISA, as recommended by the manufacturer (BD Pharmingen, San Diego, CA, USA).

### Eosinophil peroxidase (EPO) activity in lung

The cell suspensions from mouse lungs were frozen and thawed three times in liquid nitrogen. After centrifugation at 4°C for 10 min at 1000 g, the cell lysates were placed into wells of 96-well plates, followed by the addition of the chromogen and substrate solution (1.5 mmol/L of o-phenylenediamine and 6.6 mmol/L of H_2_O_2_ in 0.05 mol/L Tris–HCl, pH 8.0). After 30 minutes room temperature, the reaction was stopped with the addition of 0.2 mol/L citric acid, and the absorbance of the sample determined at 492 nm in an ELISA reader.

### Measurement of serum levels of Bt-specific IgE

Anti-Bt IgE antibody levels from mice were determined by indirect ELISA. 96-well micro titer high-binding plate (Costar, Cambridge, MA, USA) were coated with Bt (100 μg/well) overnight (at 4°C). The serum samples were added and the plates were incubated again. Biotin-conjugated IgE anti-mouse (BD Pharmingen, San Diego, CA, USA) were added to the wells and incubated during 1 hour at room temperature (RT). A solution of avidin-horseradish peroxidase (BD Pharmingen, San Diego, CA, USA) was then added to each well for 30 minutes. After that, a solution containing 3, 3′, 5, 5′-tetramethylbenzidine and hydrogen peroxide (BD Pharmingen, San Diego, CA, USA) was added and incubated during additional 30 minutes (at RT). The reaction was stopped with 4 M sulfuric acid. Between all steps the wells were washed 3 times with PBS containing 0.05% Tween 20 (PBS-T). The absorbance of each sample was determined at 492 nm in an ELISA reader.

### Lung histology

The analyses the histopathological changes and the degree of inflammation in peribronchiolar and perivascular regions were performed. The lungs were perfused, via the heart right ventricle, to remove residual blood, and immersed in 10% (v/v) formaldehyde (Sigma-Aldrich St. Louis, MA, USA). The tissue was dehydrated, embedded in paraffin and cut in 5 μm sections. The slides were stained with hematoxylin-eosin (HE) for inflammatory cell infiltration and were then stained with periodic acid-Schiff (PAS) for the evaluation of mucus production, under light microscopy with 40× magnification.

### Statistical analysis

Multiple comparisons were performed by one-way analysis of variance (ANOVA) and Tukey’s post-test (for data with normal distribution). Data were expressed as mean ± standard error of the mean. Differences in p values ≤0.05 were considered statistically significant. Each experiment was repeated at least twice.

## Results

### Quercetin is present in the methanolic extract of *Allium cepa* L

**Figure 2:**
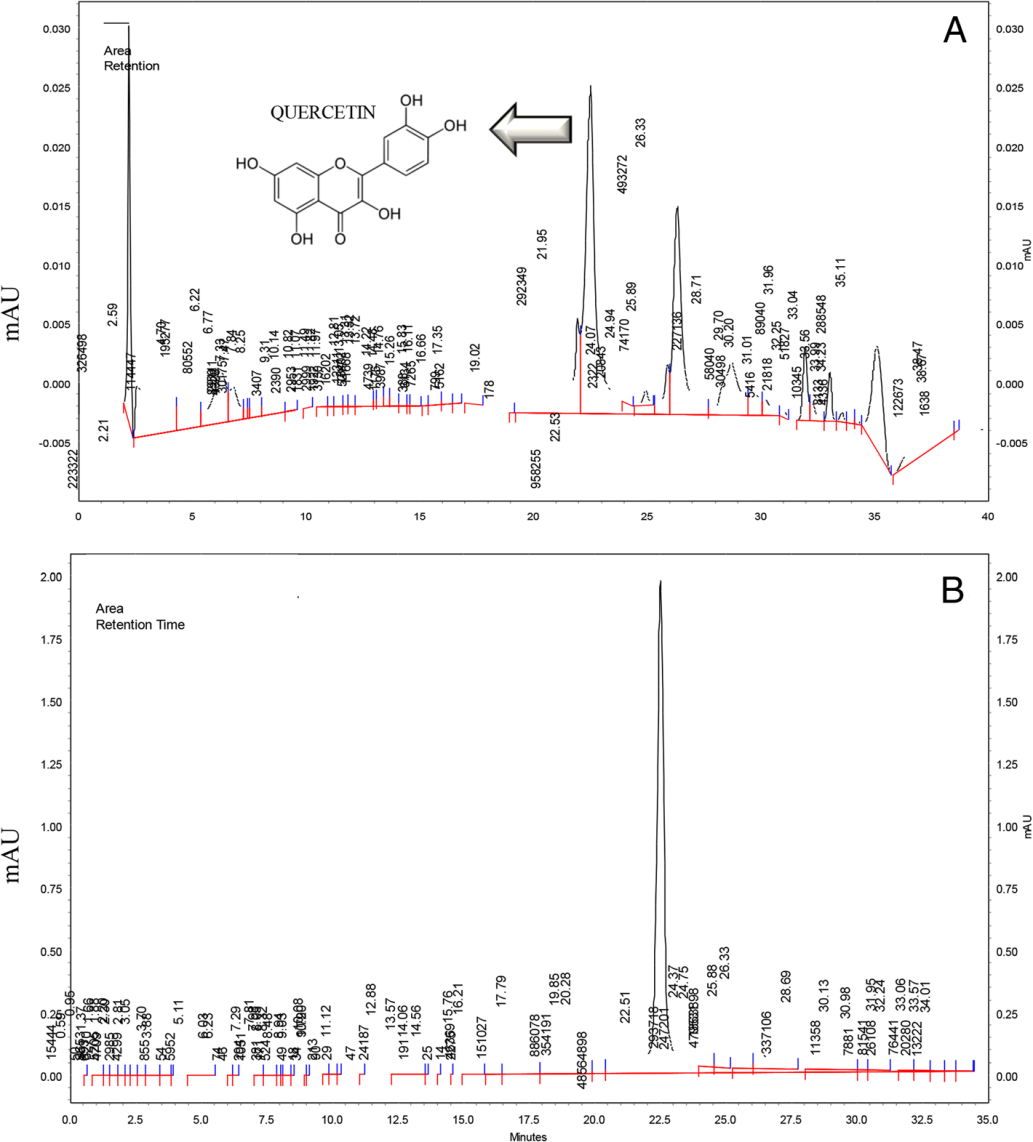
**Chromatogram of samples subjected to high performance liquid chromatography. (A)** Chromatogram of *Allium cepa* L. methanol extract. **(B)** Chromatogram of quercetin. Retention times of 22.51 minutes are shown above peaks in **(A)** and **(B)**.

The chromatogram of the methanolic extract of AcE (Figure 2A) and a quercetin standard solution (Figure 2B) demonstrated the separation of a compound in the methanolic extract with the same retention time of Qt in the standard sample (Figure 2A and B). The calculated average percentage of Qt in the AcE extract is 2.5% (based on peak area).

### Effect of AcE and Qt on cell viability and cytokine levels in spleen cells culture

**Figure 3:**
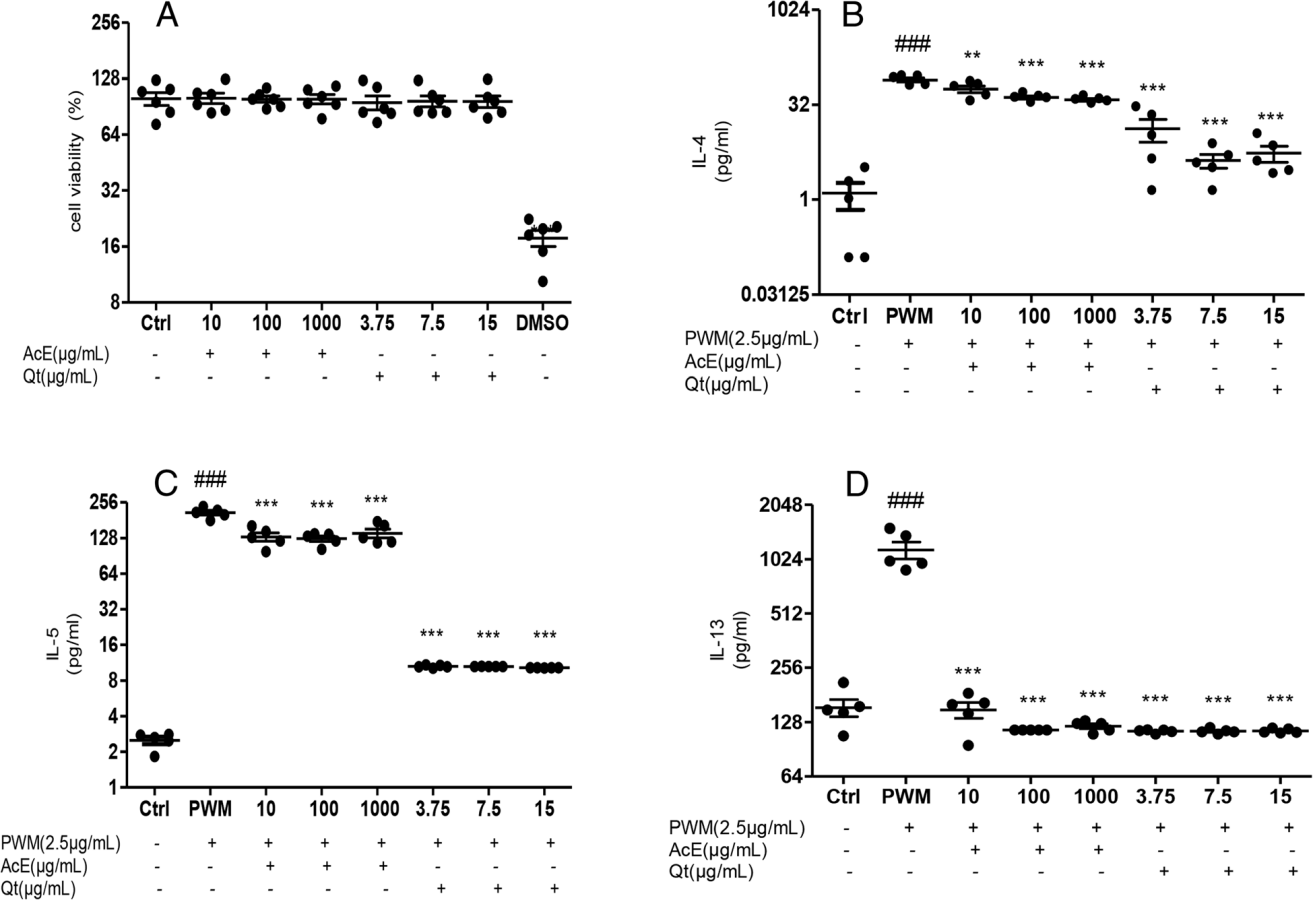
**Effect of the in vitro treatment with AcE or Qt on cell viability (A) and on the levels of IL-4 (B), IL-5 (C) and IL-13 (D).** The following groups were presented: splenocytes supernatant from animals Bt-sensitized without stimulation (control), stimulated with PWM (PWM) and stimulated with PWM and exposed to different concentrations of AcE (10 μg/ml, 100 μg/ml or 1000 μg/ml) or Qt (3.75 μg/ml, 7.5 μg/ml, or 15 μg/ml). Values represent mean ± SEM (n = 5, per group). ^###^p < 0.001 vs. control, and ^***^p < 0.001, ^**^p < 0.01 vs. PWM group (one-way ANOVA followed by Tukey’s test).

The non-cytotoxic concentrations of AcE and Qt used were determined by MTT assay. As can be seen in Figure 3A, none of the evaluated concentrations were toxic for spleen cells (AcE: 1000–10 μg/ml and Qt: 15–3.75 μg/ml). The production of Th2 cytokines, including IL-4, IL-5, and IL-13 by PWM-stimulated splenocytes from Bt-sensitized mice was increased in comparison to that by PWM-non-stimulated splenocytes from Bt-sensitized mice (p < 0.001). IL-4 (Figure 3B), IL-5 (Figure 3C), and IL-13 (Figure 3D) levels were significantly lower in the culture supernatants of splenocytes from PWM-stimulated mice that had been treated with AcE or Qt, when compared to the control group.

### Effect of AcE and Qt on airway smooth muscle contractility

**Figure 4:**
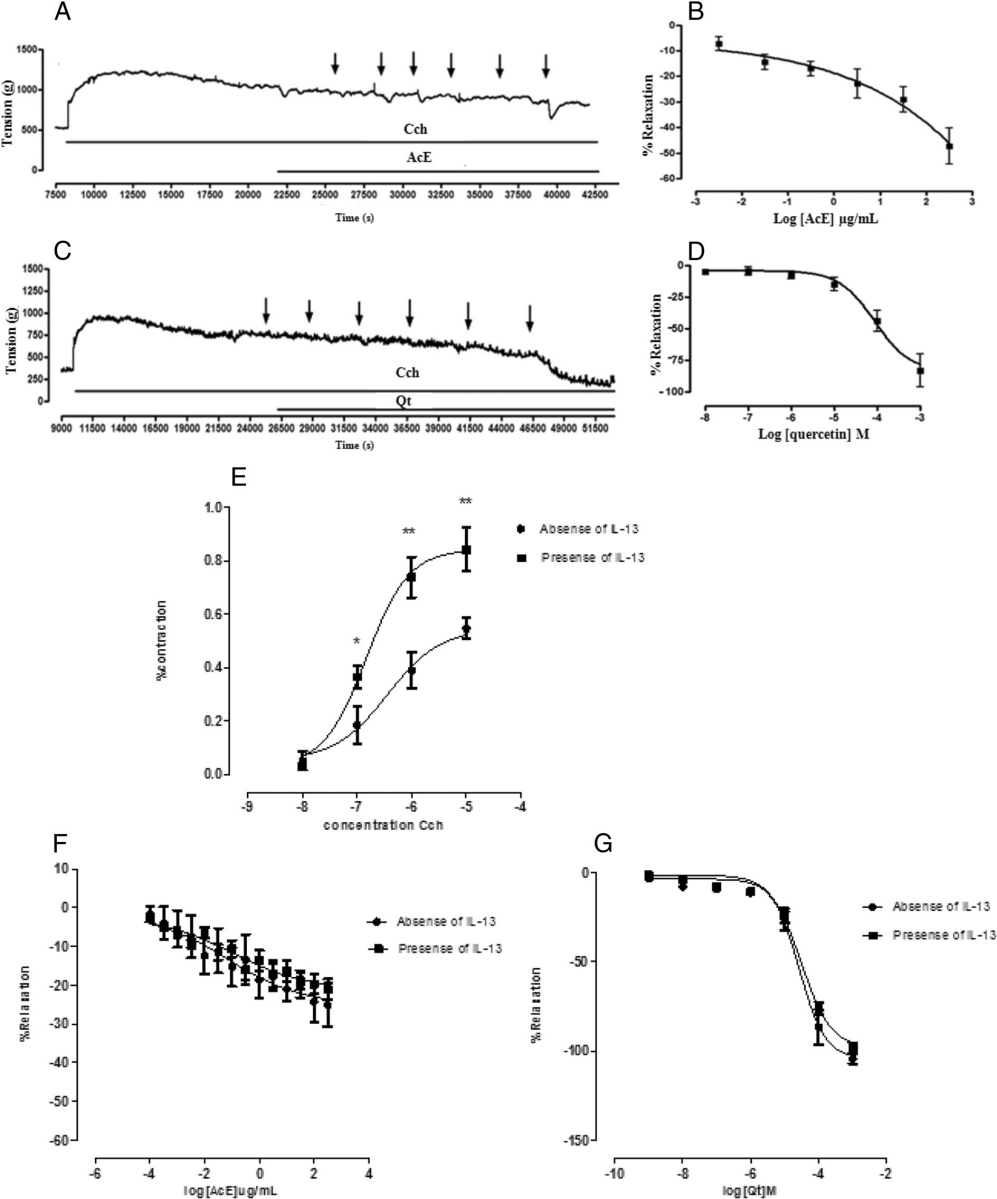
**Effect of AcE or Qt in mice airway smooth muscle. A and C)** The original record, the arrows indicate the time of addition of AcE (1, 3, 10, 30, 102 and 3 × 10^2^ /ml, cumulatively) and Qt (10^−8^, 10^−7^, 10^−6^, 10^−5^, 10^−4^ and 10^−3^ M). **B and D)** Logarithmic concentration–response curve of relaxant response of AcE (Emax = 47.2 ± 7.0 (%) EC50 = 7.1 (1.8-27.4) mg /ml) and Qt (Emax = 84.0 ± 13.1 (%), EC50 = 8.9 (4.8 to 16.2) × 10^−5^ M) on tracheal rings pre-contracted with 1 μM carbachol, in the absence of functional epithelium (n = 6). **E)** Concentration-response curve of tracheas isolated from AJ mice without epithelium pre-treated with IL-13 (■) or absence of IL-13 (•) and pre-contracted with Cch (10^−9^ M - 10^−4^ M). The concentration-response curve was significantly higher in rings pretreated with IL-13 compared with absence of IL-13. Values are expressed as means ± SEM. ^*^p < 0.05 and ^**^p < 0.01 vs. tracheas absence of IL-13. The data were examined using unpaired Student’s t-tests. **F and G)** Concentration-response curves showing the relaxant effect of AcE and Qt in tracheas rings denuded epithelium pre-contracted or not with IL-13 (n = 6).

As can be seen in Figure 4(A and C), the cumulative addition of AcE (1, 3, 10, 30, 10^2^, and 3 × 10^2^ μg/ml) and Qt (10^−8^, 10^−7^, 10^−6^, 10^−5^, 10^−4^, and 10^−3^ M), respectively, induced a transient relaxing effect, in a concentration-dependent manner, with the values of CE50 = 7.1 (1.8 – 27.4) mg/ml and CE50 = 8.9 (4.8 – 16.2) × 10^−5^ M for AcE and Qt, respectively. The data set may be seen in Figure 4(B and D), which shows an ACE concentration-response curve. The percentage of the maximum relaxation (Emax) induced by AcE was Emax = 47.2 ± 7.0 (%), and induced by Qt was Emax = 84.0 ± 13.1 (%).

Additionally, in our in vitro model of hyper-reactivity using IL-13, murine tracheal rings that were incubated overnight with IL-13 (10 ng/ml) increased the constrictor responses to Cch but without changes in pharmacological potency (Emax = 0.84 ± 0.08 (%); pD2 = 6.8 ± 0.1091) when compared with non-treated rings Emax = 0.55 ± 0.04 (%); pD2 = 6.4 ± 0.1887 (Figure 4E). Moreover, a reduction in intensity of smooth muscle contraction in the absence or presence of IL-13 with different concentrations of AcE (CE50 = 0.075 (0.011-0.5) Emax = 25.14 ± 5.52 (%)) or Qt (pD2 = 4.6 ± 0.07 Emax = 104.42 ± 2.94 (%) was observed (Figure 4F and G). However, the efficacy and potency of the tested drugs in hyper-reactive tracheal rings (sensitized with IL-13) were not altered when the drugs were tested in normal rings, not sensitized with IL-13.

### Effect of AcE or Qt on cell influx in BAL fluid and on EPO levels in lungs

**Figure 5:**
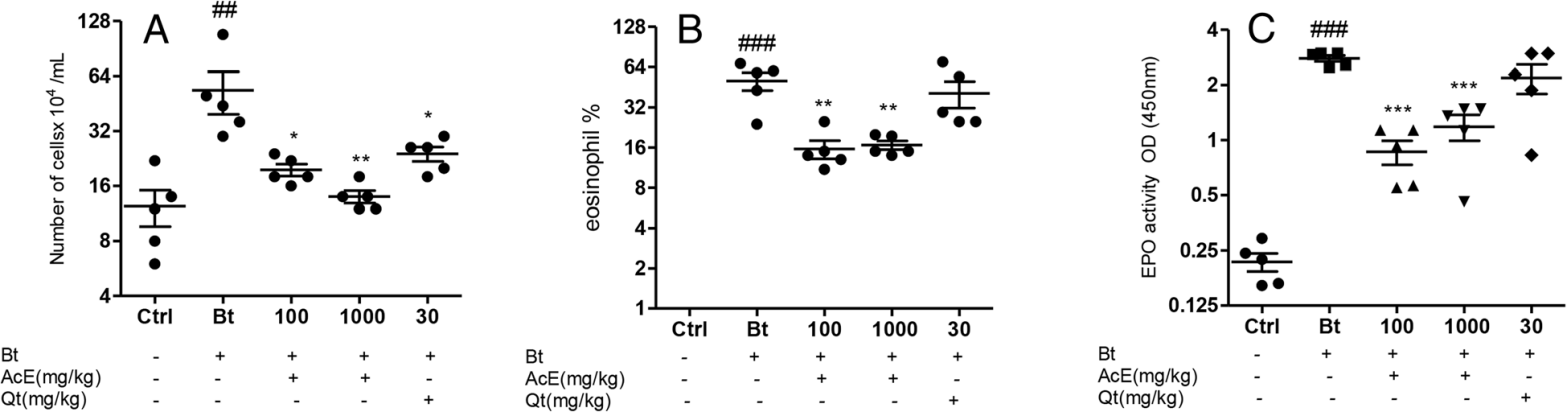
**Effect of AcE or Qt on the levels of total cells in the BAL and on EPO in lungs.** Effect of the treatment with AcE or Qt on the level of **A)** total cells counting **B)** eosinophilia in the BAL and **C)** eosinophil peroxidase (EPO) activity in lung tissue. Control (Ctrl); Bt-sensitized animals (Bt); and Bt-sensitized, AcE (100 or 1000 mg/kg) or Qt (30 mg/kg) treated mice. Values represent mean ± SEM (n = 5, per group). ^##^p < 0.01; ^###^p < 0.001 vs. control; ^*^p < 0.05; ^**^p < 0.01; ^***^p < 0.001 vs. Bt group (one-way ANOVA followed by Tukey’s test).

We examined changes in total cell numbers in the BAL fluid to determine the effects of AcE or Qt on experimental respiratory allergy. The BAL cellularity was estimated by counting the cells recruited to the BAL fluid 48 h after the last challenge. In relation to the control group, Bt-sensitized mice displayed a significant increase in total cell numbers (p < 0.01). However, in Bt-sensitized mice, the treatment with AcE_100_ (p < 0.05), AcE_1000_ (p < 0.01) or Qt_30_ (p < 0.05) decreased the total number of cells in relation to untreated Bt-sensitized mice (Figure 5A). Additionally, the lung tissue was collected 48 hours after the final Bt challenge and EPO activity were measured. The EPO levels from lung tissue of Bt-sensitized mice were increased in relation to control group (p < 0.001). The mice from Bt-sensitized group and treated with AcE_100_ (p < 0.001) or AcE_1000_ (p < 0.001) had significantly lower EPO levels after respiratory allergy induction. No effect in EPO levels was observed in mice treated with Qt_30_ (Figure 5B).

### Effect of treatment with AcE or Qt on inflammatory cell infiltration and amount of mucus in lungs

**Figure 6:**
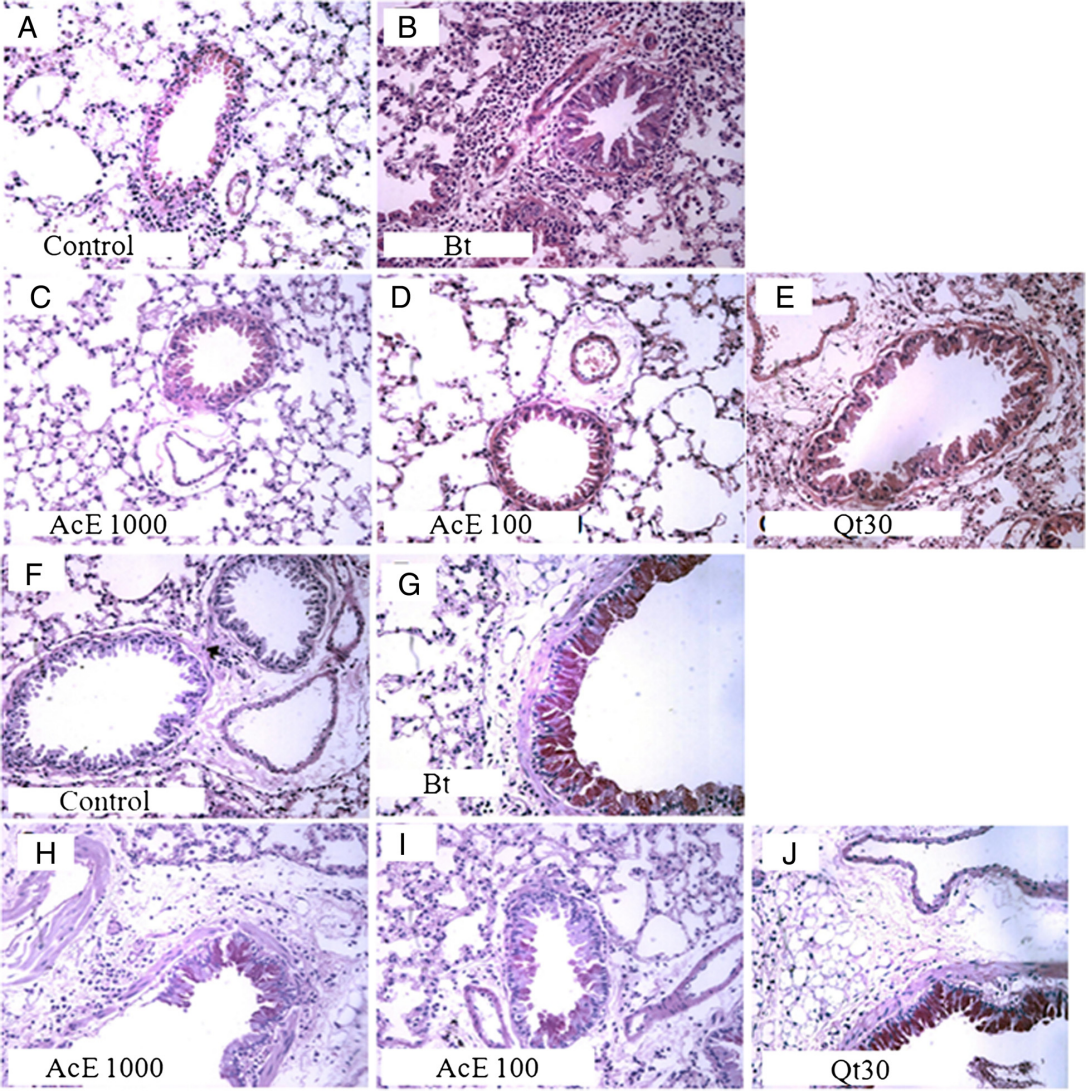
**Effect of the treatment with AcE or Qt on celular infiltration and mucus production in lung tissues.** Sections were stained with hematoxylin-eosin (magnification × 400) **(A-E)** and sections were stained with periodic acid-Schiff (magnification × 400) **(F-J)**. **(A and F)** Lung section from a control, saline-treated mice; **(B and G)** Lung section from a Bt-sensitized, saline-treated mice; **(C and H)** Lung section from a Bt-sensitized, AcE 100 mg/kg treated mice; **(D and I)** Lung section from a Bt-sensitized, AcE 1000 mg/kg treated mice; **(E and J)** Lung section from a Bt-sensitized, Qt 30 mg/kg treated mice.

Because AcE or Qt inhibits inflammatory cell recruitment into the lung tissue and EPO levels in lungs, we examined the histology of lung tissue. In Bt-induced asthmatic lung tissue, we observed a marked infiltration of inflammatory cells into the perivascular and the peribronchiolar regions (Figure 6A-E), and airway mucus hypersecretion (Figure 6F-J) compared with the normal tissue. Cell infiltration and mucus hypersecretion in lungs of Bt-sensitized were attenuated by treatment with AcE when compared with the level seen in Bt-sensitized mice, as shown in Figure 6.

### Effect of treatment with AcE or Qt on cytokine levels in BAL fluid

**Figure 7:**
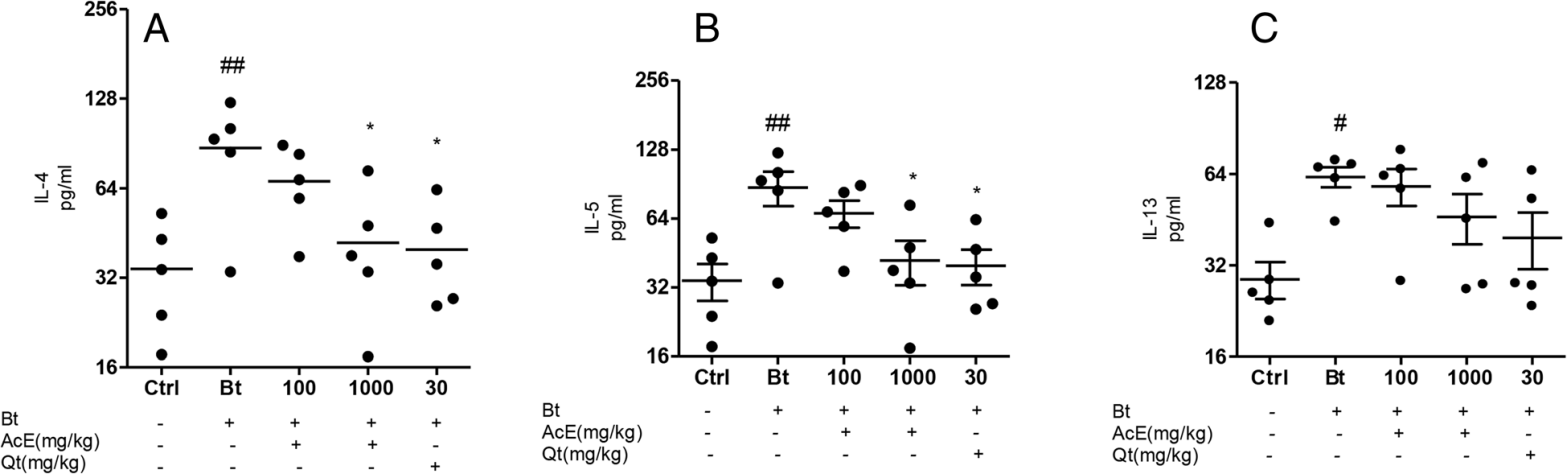
**Effect of AcE or Qt on cytokine levels in BAL fluid.** Effect of the treatment with AcE or Qt on the levels of **A)** IL-4 and **B)** IL-10 **C)** IL-13 in the BAL of Control (Ctrl); Bt-sensitized animals (Bt); and Bt-sensitized, AcE (100 or 1000 mg/kg) or Qt (30 mg/kg) treated mice. Values represent mean ± SEM (n = 5, per group). ^#^p < 0.05 vs. control; ^###^p < 0.001 vs. control; ^**^p < 0.01 vs. Bt group and ^***^p < 0.001 vs. Bt group (one-way ANOVA followed by Tukey’s test).

To determine whether AcE or Qt influenced the generation of a Th2-type immune response, cytokine concentrations in BAL fluid (IL-4, IL-5 and IL-13) were measured by ELISA 24 hours after the last challenge. As shown in Figure 7, Bt-sensitized animals had significantly higher IL-4 (p < 0.01), IL-5 (p < 0.01) and IL-13 (p < 0.05) levels in the BAL fluid than control mice. In Bt-sensitized mice the treatment with AcE or Qt significantly decreased IL-4 and IL-5 levels (p < 0.05) when compared with the levels seen in the Bt-sensitized group. The oral treatment with AcE did not affect the levels of IL-13 in the BAL of Bt-sensitized mice.

### Effect of treatment with AcE or Qt on serum levels of Bt-specific IgE antibodies

**Figure 8:**
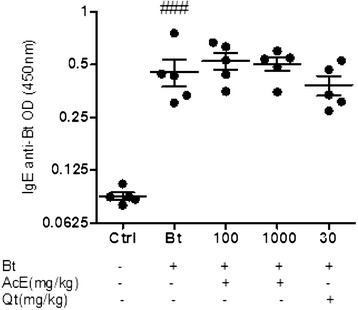
**Effect of AcE or Qt on the levels of IgE anti-Bt.** Effect of the treatment with AcE or Qt on the level of IgE in control (Ctrl); Bt-sensitized animals (Bt); and Bt-sensitized, AcE (100 or 1000 mg/kg) or Qt (30 mg/kg) treated mice. Antibody levels were measured by indirect ELISA. Values represent mean ± SEM (n = 5, per group). ^###^p < 0.001 vs. control (one-way ANOVA followed by Tukey’s test).

The cross-linking of allergen-specific IgE on the surface of mast cells upon allergen challenge is relevant to the initiation of the early asthmatic reaction. The serum levels of Bt-specific IgE were measured 24 hours after the last challenge. We observed that sensitization and challenge with Bt resulted in increased serum levels of Bt-specific IgE when compared with non-sensitized animals (p < 0.001). The treatment of sensitized mice with AcE or Qt did not reduce significantly Bt-specific IgE (Figure 8).

## Discussion

The chronic inflammatory response to allergens on allergic asthma is characterized by eosinophilia, airway hyperresponsiveness (AHR) and increased mucus production. The actions of inflammatory cells in airways are tightly regulated by a network of Th2 cytokines, such as IL (interleukin)-4, IL-5, and IL-13 [34]. The present study was conducted using a murine model of allergic airway disease induced by *Blomia tropicalis* mite that was previously characterized by our research group [29]. Previous studies show that once Bt sensitization induced cells infiltration to the lungs, increased EPO activity and high amounts of Bt-specific IgE were obsrved. All these were associated to a Th2-type cytokine profile (with the production of IL-5, IL-13 and IL-4), which is important for the development and maintenance of the Th2 response characteristic of asthma [29,15]. The resolution of inflammation is fundamental to the return of normal physiological parameters. In this study we observed that *Allium cepa* L. and quercetin were associated to anti-inflammatory and bronchodilator activities in an airway inflammation murine model of asthma. As previously described [29], the total BAL cell numbers were increased in Bt-sensitized mice. Additionally, there was a statistically significant decrease in total cell numbers in the groups of Bt-sensitized animals treated with AcE_10_, AcE_1000_, or Qt_30_. Drugs that modulate the recruitment of eosinophils and their activation may be important in reducing lung inflammation in asthma [35,29,36]. AcE and Qt when administered to allergic mice induce a decrease in total cellularity in BAL, a parameter required to suppress eosinophil degranulation. Despite the fact that Qt_30_ did not significantly modulate EPO in the lung, the Bt-sensitized mice treated with AcE_100_ or AcE_1000_ had significantly lower EPO levels than untreated Bt-sensitized control mice, suggesting that a substance other than Qt is mediating this effect in AcE.

Previous studies in humans have shown that the addition of anti-IL-5 monoclonal antibody on asthma therapy significantly accelerated apoptosis of eosinophils, thereby decreasing pulmonary eosinophilia [37]. In this study, we could observe a significant reduction of IL-5, accompanied by a significant decrease in eosinophil peroxidase in the lungs of sensitized mice treated with Bt and AcE.

AcE and Qt had a statistically significant inhibitory effect on IL-4 and IL-5 levels in the BAL fluid of mice treated with AcE_100_ and Qt_30_. Despite the modulation of IL-13 *in vitro*, a similar effect could not be observed *in vivo*. AcE and Qt suppressed the secretion of IL-4, IL-5 and IL-13 by spleen cells stimulated with PWM. This fact reinforces the importance of *in vivo* studies to elucidate the mechanisms involved in drug activities.

IL-4 along with IL-5 modulates eosinophil activation to stimulate B cells, IgE production, and mast cell degranulation [38] and their reduction, has been shown in this study, could be involved in the modulation of symptoms of allergic disease.

IL-13 also plays important role in allergic asthma [33,34], such as eosinophilic lung infiltration and mucus hypersecretion [39,40], which could be observed in our allergic mice. As there was no significant modulation of IL −13 by the tested drugs, we believe that in this work, as well as in other prior work, mucus is strongly connected to increased levels of IL-13 on asthmatic mice [40]. Nevertheless, AcE (AcE_100_) was able to decrease the amount of mucus in the lungs of treated and Bt-sensitized mice. This effect was not observed with Qt_30_.

Regarding IgE levels, no reduction on antibody titers were observed. These results may be related to the lack of a modulating effect on IL-13, which in addition to IL-4, is an important regulator of IgE production. Another hypothesis to explain the absence of effect on IgE levels is related to the short duration of our acute model, which may not be suitable for evaluating humoral responses, considering the necessary time for the variation in certain cytokine levels to reflect on serum antibody concentrations.

The interaction between the allergen and IgE present on mast cells plays a critical role in allergic inflammation. IgE cross-linking on mast cells leads to the release of histamine, prostaglandin (PG) D2 and leukotrienes, which results in smooth muscle contraction, mucous secretion and vasodilatation [41,42]. In previous studies [25,27,28], the therapeutic potential of flavonoids in isolated trachea was investigated. In this study it was observed that both AcE and Qt exerted a relaxing activity on the smooth muscle of isolated murine trachea precontracted with Cch. Additionally, our study supports the findings that interleukin-13 has been implicated as a key cytokine in smooth muscle hyperreactivity on asthma, since we demonstrated an increased contractility in response to Cch in airway smooth muscle induced by IL-13 [33]. Significant changes in Emax were observed, indicating that IL-13 seemed to increase smooth muscle contractility and cause hyperreactivity at the level of contractions. However the bronchodilatory potential of AcE or Qt is not selective for IL-13-induced hyperactivity pathway, as there was no difference between their effect on hyperreactive smooth muscle (IL-13- sensitized) and their effect on normal smooth muscle.

This study corroborates other previous studies on the anti-inflammatory and antiallergic properties of *Allium cepa* L. and quercetin [16-19,25], which might be linked to the ability of AcE an quercetin in down-modulate inflammatory processes through different signaling pathways such as NF-κB [43-45]. On the other hand, some authors discuss allergic reaction to onion. In such previous studies regarding onion hypersensitivity, authors reported patients producing IgE as well as cell-mediated mechanisms against plant lipid transfer proteins (LTPs) [46]. These are a group of highly-conserved proteins found in higher plant tissues [47]. The hypersensitivity to onion has been described as a cause of asthma induced by handling of onions. However, few publications in the literature report allergic reactions due to onion ingestion despite its wide use [48]. No toxic effect was found in animals exposed to AcE in the present study (data not shown).

AcE displayed a greater efficacy when compared to Qt, as it was able to regulate a greater amount of the parameters evaluated. This research corroborates with previous studies of natural products, which state that better biological responses can be achieved by the additive or synergistic effects of different compounds from an extract [49]. However, further studies are required in order to elucidate the cellular and molecular mechanisms by which AcE performs its action and to determine what substances are present in AcE in addition to quercetin, that contribute to the observed biological responses.

## Conclusions

The results obtained in this work provide the first evidence that *Allium cepa* L. may have an anti-allergic effect more intense than Qt, and may be a future target for new molecules to treat allergic asthma. Our work may also validate and explain the long-held traditional use of this species by folk Brazilian medicine to treat asthma. This work also opens new perspectives in the context of elucidating the cellular and molecular mechanisms involved in the mechanism of action of *Allium cepa* L. as a way to enable clinical trials to evaluate its efficacy in humans.
